# Plasma membrane imaging with a fluorescent benzothiadiazole derivative

**DOI:** 10.3762/bjoc.15.257

**Published:** 2019-11-06

**Authors:** Pedro H P R Carvalho, Jose R Correa, Karen L R Paiva, Daniel F S Machado, Jackson D Scholten, Brenno A D Neto

**Affiliations:** 1 Laboratory of Medicinal and Technological Chemistry, University of Brasília, Chemistry Institute (IQ-UnB), Campus Universitário Darcy Ribeiro, Brasília, Distrito Federal, 70904-970, Brazil; 2 Laboratory of Molecular Catalysis, Institute of Chemistry, Graduate Program (PPGQ), Universidade Federal do Rio Grande do Sul, Porto Alegre, RS, 91501-970, Brazil

**Keywords:** benzothiadiazole, bioprobe, cell imaging, fluorescence, mitochondria, molecular architecture, plasma membrane

## Abstract

This work describes a novel fluorescent 2,1,3-benzothiadiazole derivative designed to act as a water-soluble and selective bioprobe for plasma membrane imaging. The new compound was efficiently synthesized in a two-step procedure with good yields. The photophysical properties were evaluated and the dye proved to have an excellent photostability in several solvents. DFT calculations were found in agreement with the experimental data and helped to understand the stabilizing intramolecular charge-transfer process from the first excited state. The new fluorescent derivative could be applied as selective bioprobe in several cell lines and displayed plasma-membrane affinity during the imaging experiments for all tested models.

## Introduction

The selective staining of plasma membranes is of paramount importance to study cellular processes and events associated with this dynamic cellular component. After more than one century of the idea considering cell plasma membranes as lipid bilayers [1], the importance to understand the functions, processes and events associated with plasma membranes is still vital. We are only beginning to understand many of the processes and functions related to this component responsible for the boundaries of the cells [2–4]. Various details have emerged due to the development of new sensitive molecular probes capable of staining organelles and cell components selectively, however, many open questions remain.

Plasma membranes are the natural barrier between the extracellular environment and the cytoplasm, thus playing a pivotal role in cellular uptaking processes, trafficking and signaling [5]. Many models aim at describing the membranes’ behavior in several solvents and aqueous solutions [6]. However, the direct imaging and observation of plasma membranes in live cells is still one of the most promising strategies to investigate their roles, functions and to relate them with specific cellular responses [7].

The selective imaging of plasma membranes allows the tracking of the cell morphology, the cell status, cellular division step, signal transduction, apoptosis and even necrosis [8–10]. The monitoring of plasma membranes, their biophysical properties, endocytosis/exocytosis of several types of molecules, as well as their dynamic changes, may be performed by using fluorogenic organic dyes or derivatives thereof [11–15].

The design and synthesis of small organic fluorescence imaging probes capable of selectively stain plasma membranes has been proven, however, often as a challenging task. The development of small organic probes, in general, has been hindered by the requirement of multistep syntheses to obtain the fluorophore and by poor performances related to most of them. Therefore, many studies are still based on the use of WGA [16] (wheat germ agglutinin) or membrane proteins bearing fluorescent protein tags [11]. Water solubility is another issue that has to be considered. For the development of new fluorogenic dyes for plasma membrane imaging there are two desirable features of the product to be considered: solubility in aqueous media and affinity for membranes. However, based on the available reports these properties seem to be antagonistic. Breakthrough works have, however, described the successful design and application of water-soluble plasma membrane probes [17–18].

**Figure 1:**
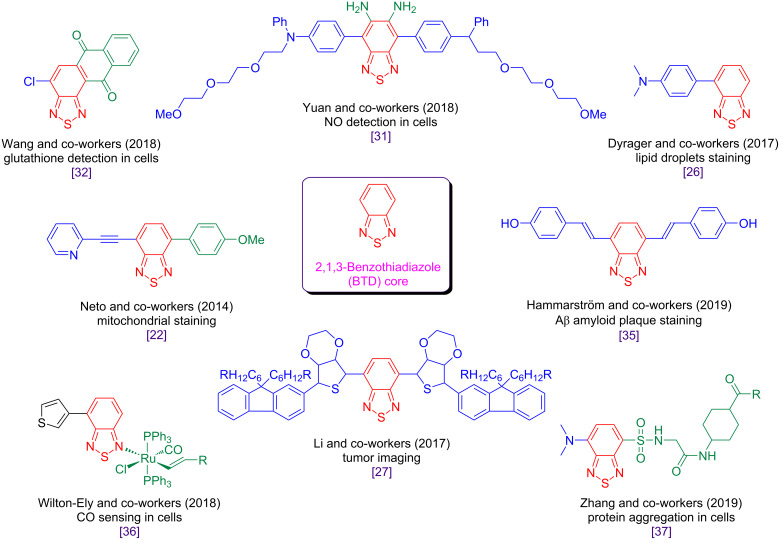
The 2,1,3-benzothiadiazole (BTD) core and its derivatives that are successfully applied in bioimaging experiments.

We have been developing a new class of selective bioprobes based on the derivatization of the 2,1,3-benzothiadiazole (BTD) core (Figure 1) [19–22]. After we disclosed the use of these BTD derivatives as a new class of selective fluorescence imaging probes, many contributions [23–37] appeared successfully applying fluorescent BTDs in bioimaging experiments (see examples in Figure 1).

Based on our interest in the development of new bioimaging agents [38–40], we disclose herein the design, synthesis, properties and application of a new fluorogenic BTD derivative as a water-soluble selective plasma membrane probe for bioimaging experiments.

## Results and Discussion

**Scheme 1:**
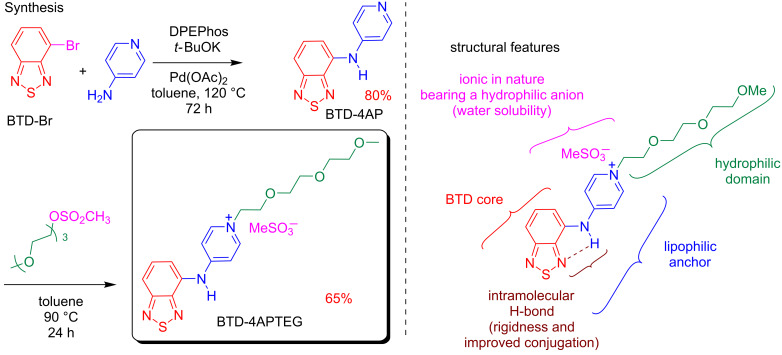
Synthesis of the plasma membrane BTD probe (BTD-4APTEG) and its structural features.

The new water-soluble fluorescent BTD derivative (named BTD-4APTEG) was planned and synthesized as shown in Scheme 1. The new fluorescent structure is accessible in a two-step procedure from the commercially available 4-bromo-2,1,3-benzothiadiazole (BTD-Br) and 4-aminopyridine (4AP), as we have recently described [41]. The Buchwald–Hartwig amination protocol afforded the fluorescent BTD-4AP in 80% yield after purification [41]. The new derivative was then synthesized by a direct alkylation reaction which afforded the desired compound BTD-4APTEG in 65% yield after purification (see details in the Experimental section).

**Figure 2:**
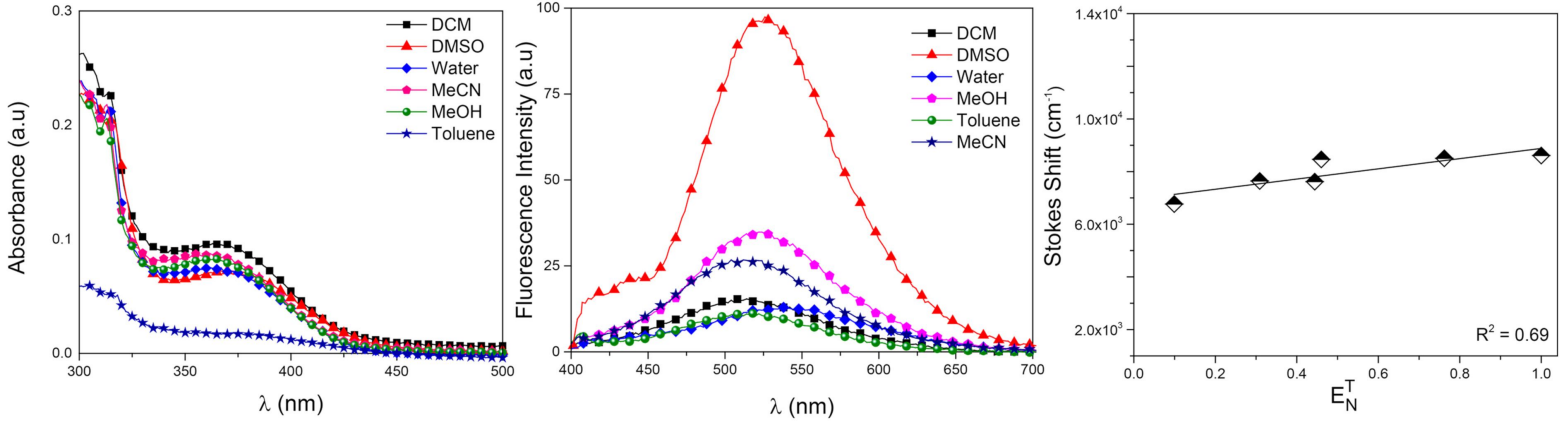
(Left) UV–vis, (center) fluorescence emission and (right) solvatochromic effect (Stokes shift in wavenumbers versus solvent polarity in ETN) of the synthesized BTD-4APTEG (10 μM for all analyses).

The structure of BTD-4APTEG bears a lipophilic anchor to improve its affinity towards the lipidic bilayer of the plasma membrane. In fact, the lipophilic character of similar small BTD derivatives demonstrated improved affinity for lipid-based structures, as we [42–43] and others [26,29] have shown. The intramolecular H-bond is in addition responsible for the rigidity of the structure and enables a better conjugation with the 4AP substituent at the C4 carbon of the BTD heterocyclic core. The ionic nature of the structure in combination with a hydrophilic anion (MeSO_3_^−^, methanesulfonate) and the presence of the hydrophilic domain (triethylene glycol monomethyl ether) make the dye a water-soluble BTD derivative. The photophysical properties of the new compound BTDE-4APTEG have been investigated and the results are summarized in Table 1 and presented in Figure 2.

**Table 1:** UV–vis and fluorescence emission data (in different solvents at 10 μM for all analyses) for the synthesized compound.

Compound	Solvent	λ_max,abs_ (nm)	log ε	λ_max,em_ (nm)	Stokes shift (nm/cm^−1^)

BTD-4APTEG	CH_2_Cl_2_	368	3.0	512	144/7643
	DMSO	375	2.9	525	150/7619
	MeCN	363	2.9	518	155/8243
	MeOH	362	2.9	523	161/8504
	toluene	383	2.2	517	134/6767
	water	370	2.9	543	173/8611

ϕ_toluene_ 0.002, ϕ_MeCN_ 0.02, ϕ_water_ 0.01, ϕ_DCM_ 0.02, ϕ_MeOH_ 0.03, ϕ_DMSO_ 0.02.

All absorption maxima were observed in the UV region close to 375 nm and with reasonable molar extinction coefficients. Large Stokes shifts were noted in all tested solvents (134–173 nm), thus pointing to efficient stabilizations through intramolecular charge-transfer (ICT) processes from the excited states. The largest Stokes shift was noted in the aqueous solution, indicating the dye’s stability in this solvent. The solvatochromic analyses of ETN vs Stokes shifts using the values provided by Richardt [44] were found in accordance with the ICT proposition and the calculated linear correlation from the plots [45] corroborated this proposition. The photostability of the new compound was measured in aqueous media as a preclude of the bioimaging experiments and proved to be stable under constant light irradiation for more than 4 hours (see Figure S4 in Supporting Information File 1).

Theoretical calculations were then performed for a better comprehension of the photophysical data obtained for BTD-4APTEG by means of the time-dependent density functional theory (TD-DFT). In practice, when applying DFT calculations, there is no “universal” exchange correlation functional (XCF), thus the performance of different XCFs in simulating the absorption spectra of BTD-4APTEG had to be assessed. We aimed at describing the maxima absorption peak position associated with the π–π* transitions, as expected for this type of 4,7-disubstituted BTDs [46–50].

**Figure 3:**
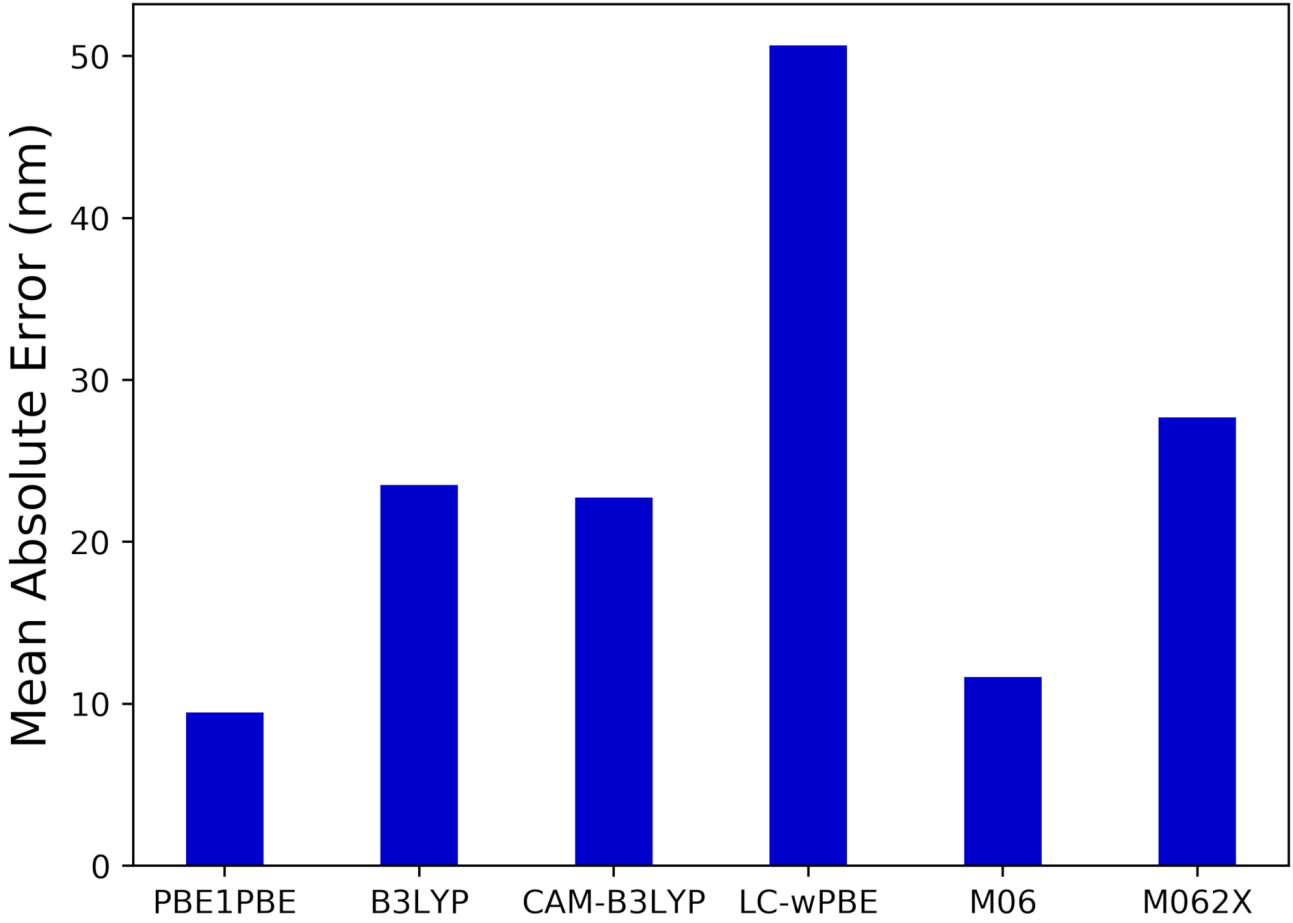
Mean absolute error (MAE) comparing both the experimental and the estimated TD-DFT λ_max_ positions in different solvents for BTD-4APTEG. The computed λ_max_ value corresponds to the largest wavelength band associated with the S_0_ → S_1_ electronic excitation.

In Figure 3 the mean absolute error (MAE) between the theoretical and experimental absorption maxima (λ_max_) in different solvents is shown. The results showed the hybrid XCF, PBE1PBE yielded the best overall performance across all studied solvents with an absolute deviation below 10 nm. PBE1PBE was therefore selected as the most suitable XCF (amongst the six investigated XCFs) for studying the photophysical properties of BTD-4APTEG.

**Figure 4:**
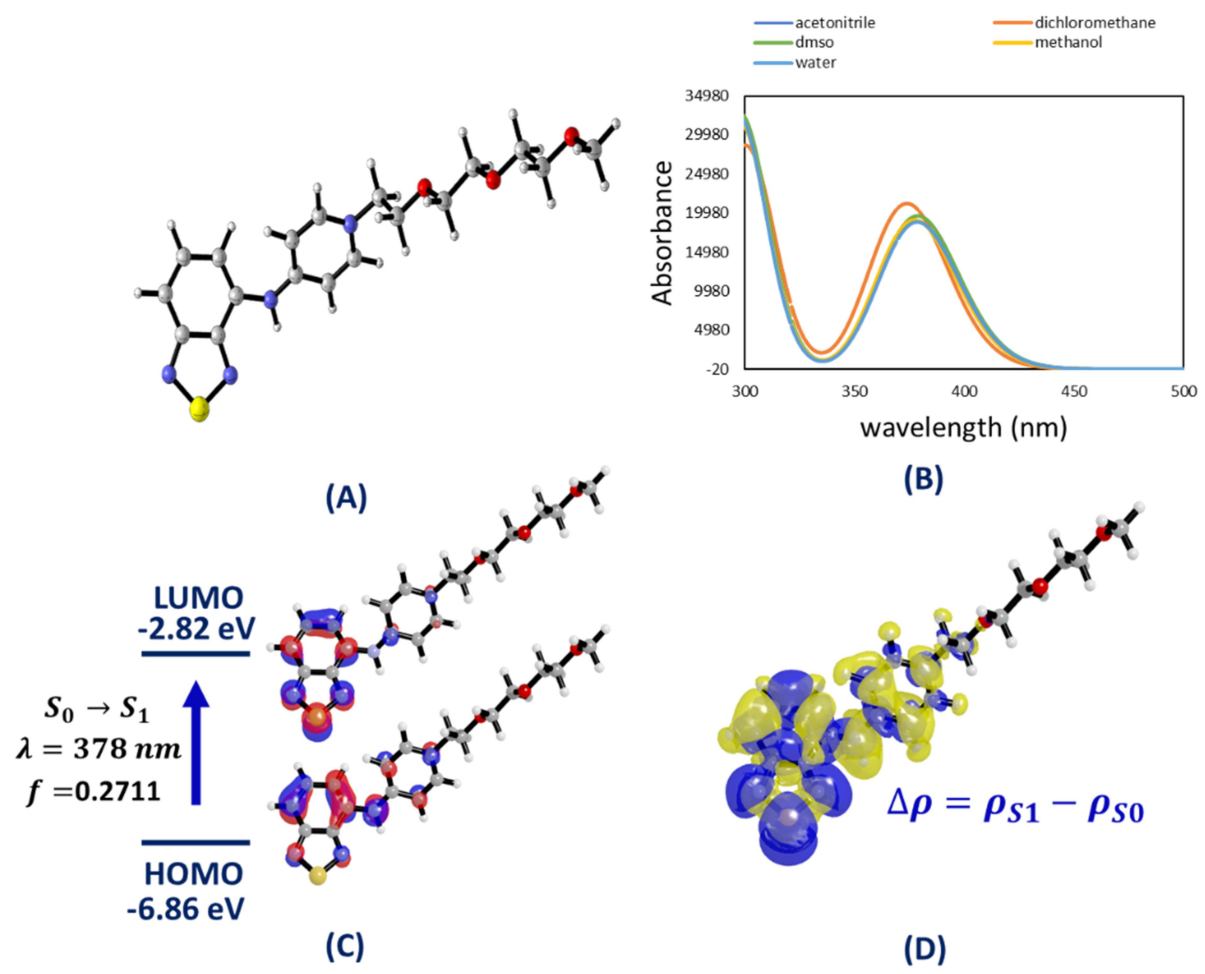
(A) CAM-B3LYP/6-311+G(d) optimized geometry of BTD-4APTEG (implicit DMSO). (B) TD-DFT UV–vis spectra of BTD-4APTEG calculated at the PBE1PBE/6-311+G(2d,p) level of theory. Solvent effects were estimated under the IEF-PCM formalism. (C) PBE1PBE/6-311+G(2d,p)//CAM-B3LYP/6-311+G(d) HOMO and LUMO orbitals of BTD-4APTEG in DMSO involved in the S0→S1 electronic excitation associated with the longest wavelength band. (D) Electron density difference between the first excited and ground states (Δρ = ρ_S1_ – ρ_S0_), where blue regions correspond to density accumulation whereas the yellow means the density depletion.

The structure geometry was fully optimized in acetonitrile, dichloromethane, DMSO, methanol and water (Figure 4A). It turned out that the optimized geometries were not affected by the implicit solvation model affording root mean squared deviations of atomic positions of about 10^−4^ Å. The obtained CAM-B3LYP/6-311+G(d) geometries showed that the BTD core of the BTD-4APTEG is twisted by nearly 30° with respect to the NH fragment (see Figure S5 in Supporting Information File 1). Although this torsion diminishes the strength of the H-bond and conjugation, the effect is not strong enough to affect the emissive properties of the designed structure, as noted for the calculated properties of the designed structure (Figure 4).

The UV–vis absorption spectra of BTD-4APTEG calculated at the PBE1PBE/6-311+G(2d,p)//CAM-B3LYP/6-311+G(d) are shown in Figure 4B and solvent effects were also accounted using the IEF-PCM formalism [51]. The theoretical absorption bands showed an excellent agreement with those obtained experimentally in both terms the peak positions as well as their relative intensities.

The π–π* band is entirely described by a HOMO–LUMO vertical transition as visualized in Figure 4C. The LUMO orbital is strictly distributed over the BTD core in the BTD-4APTEG molecule, which is the net result of its electron density withdrawal character. The HOMO orbital distributes over the BTD basic scaffold and the vicinal ring but does not involve participation of the side chain of the chromophore. The electron density difference between the ground S_0_ and first excited state S_1_ (∆ρ = ρ_S1_ − ρ_S0_), as shown in Figure 4D, highlights the directionality of the electron density transfer with a great deal of ICT between the cationic heterocycle and the BTD core, which stabilizes the excited state of the synthesized chromophore. These theoretical results agreed well with the experimental data obtained for the newly developed dye.

DFT calculations were also employed to forecast the lipophilic character of the dye utilizing several XCFs. Experimentally, lipophilicity can be measured by means of the logarithm of the *n*-octanol/water partition coefficient (known as log *P*_ow_), which is defined as the equilibrium concentration ratio of the analyte distributed between these two phases [52]. The theoretical logarithm of the partition coefficient for the water/*n*-octanol mixture at both constant temperature and pressure was computed using Equation 1 [53]

[1] $$\log K_{ow}=\frac{\Delta G_{\text{water}}^{\text{solv}}-\Delta G_{n\text{-octanol}}^{\text{solv}}}{2.303RT}$$

calculated at 298 K.

Table 2 summarizes the solvation free energies of BTD-4APTEG in water and *n*-octanol solutions and the log *K*_ow_ obtained with the solvation model based on solute electron density (SMD) [54]. The DFT calculations qualitatively returned a preference for nonpolar environments only when B97D3 (GGA level) and ωB97XD (long range-corrected hybrid level) XCFs were employed. Both B97D3 and ωB97XD were strongly recommended by a thoroughly benchmarking of DFT methods for thermochemistry by Goerik and Grimme [55].

**Table 2:** Calculated solvation free energies in water (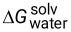), in 1-octanol (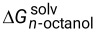) and corresponding partition coefficient log *K*_ow_ for BTD-4APTEG. Solvent effects included with the SMD solvation model.^a^

XCF	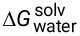	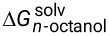	log *K*_ow_

B3LYP	−58.70	−57.12	−1.160
B97D3	−54.92	−55.38	0.333
M11	−58.74	−56.91	−1.341
M06-2X	−57.92	−56.18	−1.272
ωB97XD	−58.12	−59.90	1.305
PW6B95	−56.66	−56.57	−0.800
PW6B95-D3	−57.90	−56.81	−0.792
PBE1PBE	−58.89	−58.84	−0.029

^a^All energetic values expressed in kcal mol^−1^.

**Figure 5:**
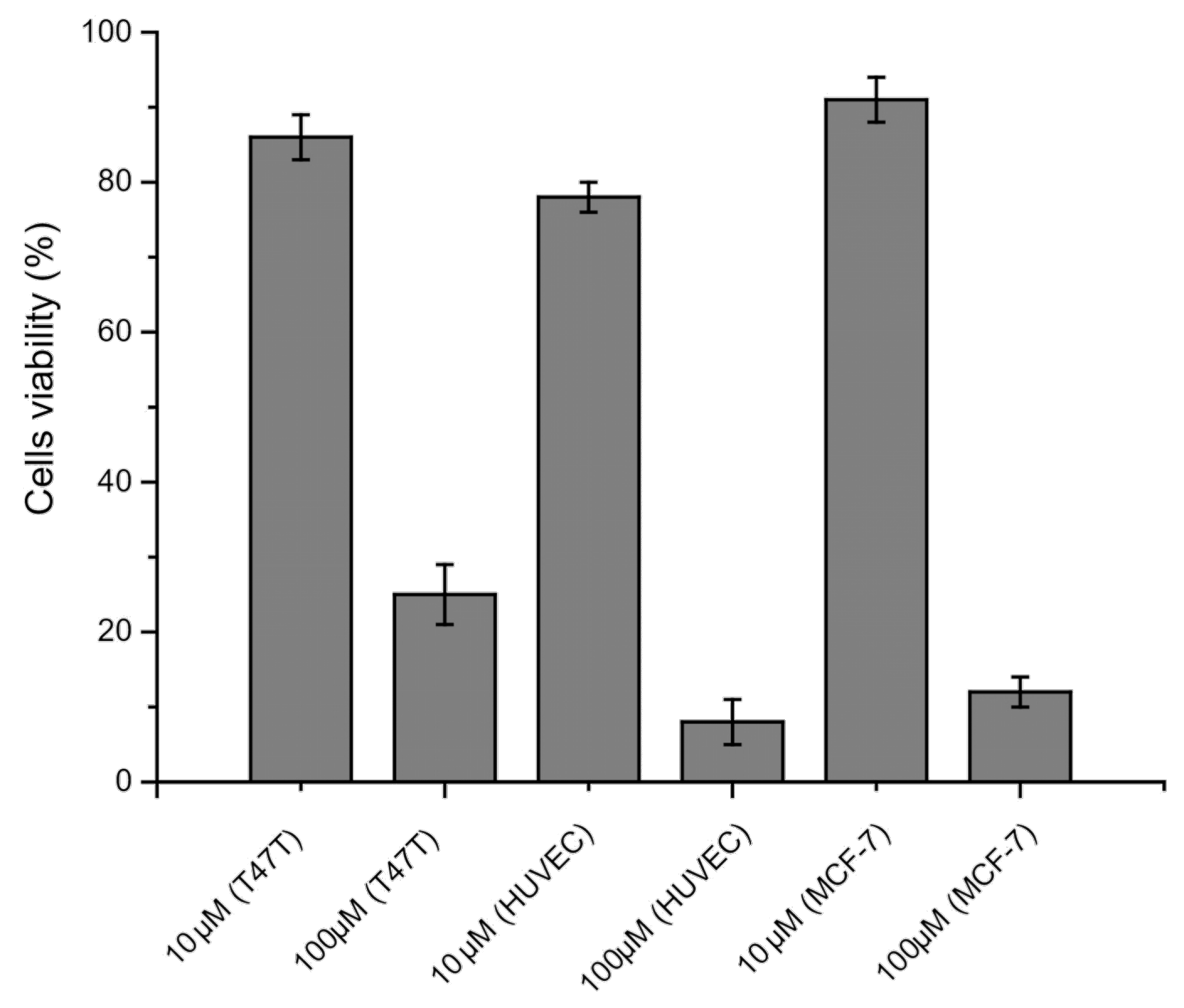
Cellular viability determined by MTT analysis after 24 h treatment with the developed dye BTD-4APTEG. No statistically significant cytotoxic effect was observed after 24 h incubation with the new dye BTD-4APTEG at 10 μM. However, the dye induced strong cytotoxic effects in all tested cell lines at 100 µM (*p* < 0.05).

The new dye was then submitted to an MTT assay to investigate possible cytotoxicity effects and the concentrations to use the probe without causing any harm to the cells (Figure 5). Only at concentrations up to 100 μM cytotoxic effects were noted and at 10 μM no effect was observed for the designed BTD-4APTEG. For the subsequent bioimaging experiments, the fluorophore was tested at 1 μM, that is at a concentration 100-fold lower than that of the cytotoxic effect.

**Figure 6:**
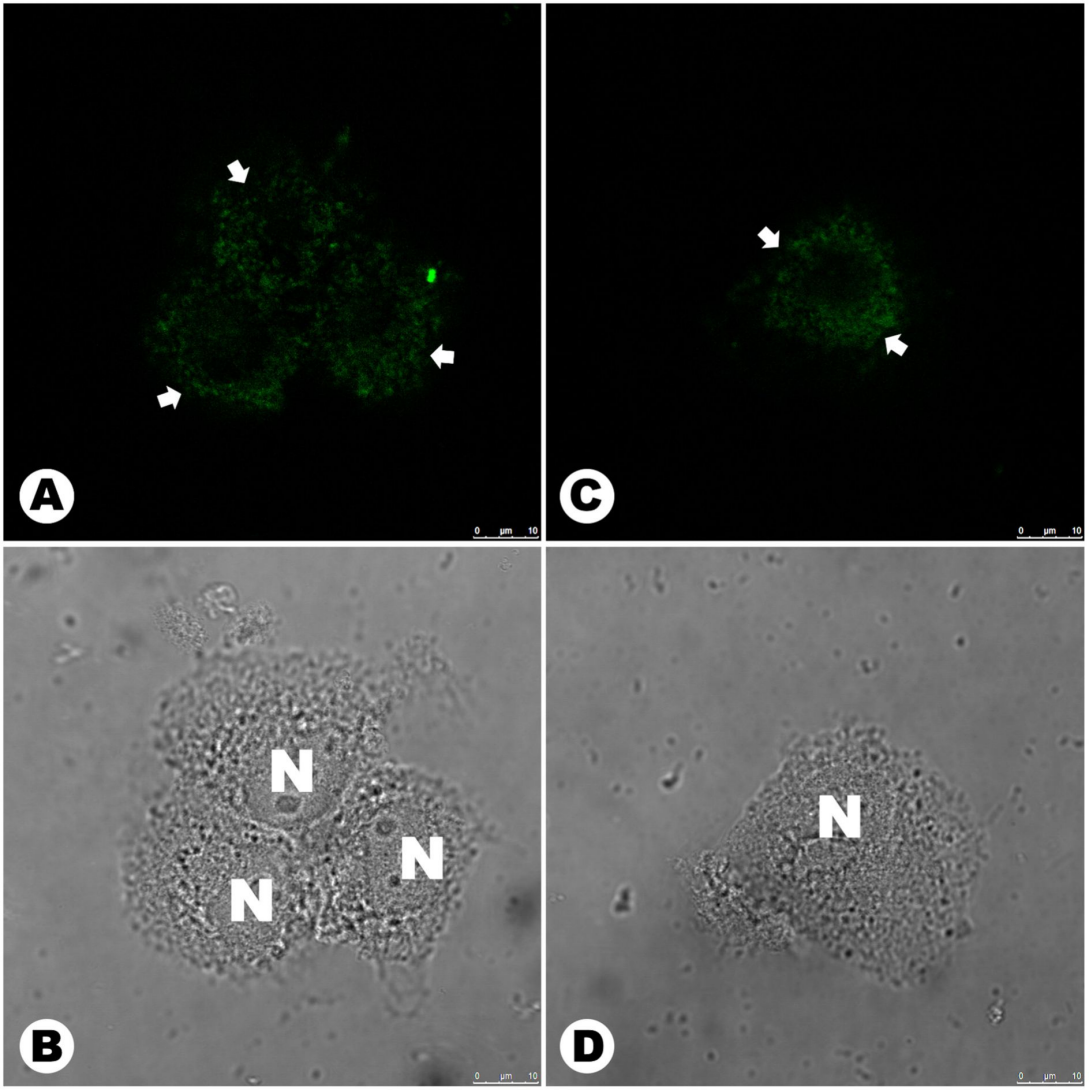
MCF-7 cells incubated with BTD-4APTEG (1 μM) in live (A) and (B) and fixed cells (C) and (D). (A) and (C) show the staining distribution of the new dye BTD-4APTEG in the plasma membrane of the cells. (B) and (D) show the normal morphological aspects of the samples by phase contrast microscopy. The dye was found accumulated in the peripheral region of the cellular membrane (white arrows) in both samples. The letter N indicates the nuclei of the cells (scale bar of 10 μm).

The new compound was then tested as bioimaging probe in live and fixed cells (Figure 6). As can be seen, the green fluorescent dye BTD-4APTEG was found most concentrated at the plasma membranes of the MCF-7 cells in both, live and fixed cells (Figure 6A,C), and not in the cytosol, thus pointing firmly to its affinity for the plasma membrane.

**Figure 7:**
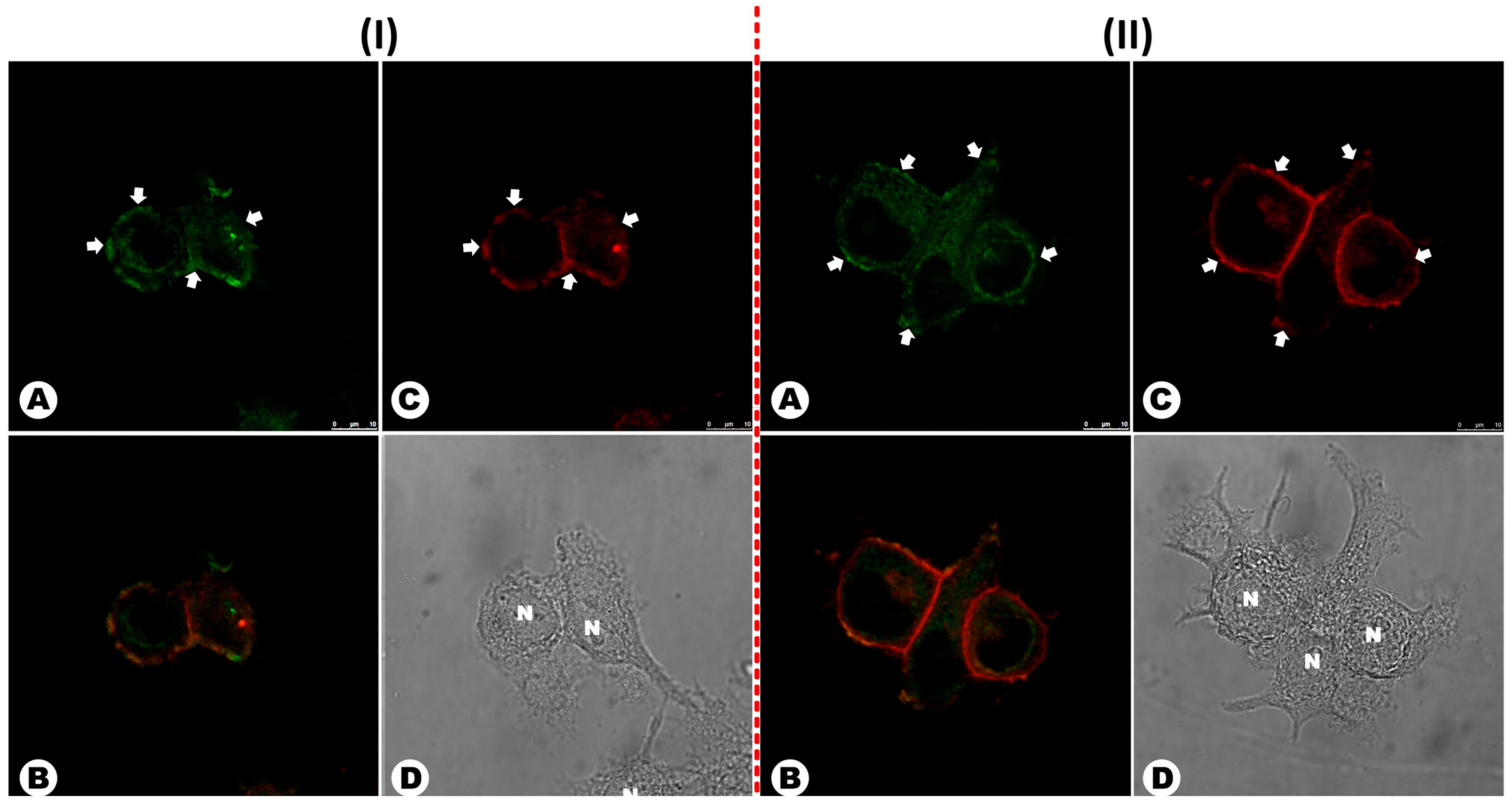
Co-staining experiments using the commercially available CellMask (red emission) and BTD-4APTEG (green emission) in (I) live and (II) fixed MCF-7 cells. (A) Plasma membranes stained with the designed BTD-4APTEG. (B) Overlay of (A) and (C) showing the orange emission as the result of both green and red emissions. (C) Plasma membranes stained with the commercially available CellMask. (D) Shows the normal morphology of the cells by phase contrast microscopy. Arrows indicate the peripherical accumulation of the dyes in the plasma membranes. The letter N indicates the nuclei of the cells (scale bar of 10 μm).

To confirm the selectivity of BTD-4APTEG towards the plasma membrane of both live and fixed cells, co-staining experiments using the commercially available probe known as CellMask were also conducted and the results are shown in Figure 7. Both bioprobes were found in the plasma membranes of the cells and their superposition (Figure 7B showing the images overlay) afforded an orange emission (green plus red).

Although there was no doubt regarding the preference of BTD-4APTEG for the plasma membranes, a Pearson correlation coefficient (PCC) [56–58] analysis between the two fluorescent signals (green and red emissions) from BTD-4APTEG and from CellMask, was performed using ten independent analyses of ten different images. The quantitative PCC showed an agreement of 79% and 77% for live and fixed cells, respectively (see Figures S6 and S7 in Supporting Information File 1). The quantitative results obtained by PCC validate the qualitative analysis shown in Figure 7, therefore supporting the dye’s affinity for the plasma membranes. The negative control was performed using BTD-4APTEG 90° counterclockwise rotation [56–58]. The analyses also provided evidence that no random colocalization was taking place (Figures S6 and S7 in Supporting Information File 1). It is known that CellMask is capable of entering the cells to some extent, especially when using fixed cells. Thus its selectivity seems to be smaller than the one observed for the new green emitter.

Although the plasma membrane separates the interior of the cell from the extracellular environment, there is a massive material transfer between both sides [59]. These materials typically are transported by vesicles which assemble large organelles within the cell cytoplasm called endosome. There are at least two different endosome types (i.e., early and late endosome) and one of them is localized near to the plasma membrane (early endosomes) whereas the other is found near to the nucleus (late endosomes) [60–62]. These organelles are formed by plasma membrane invaginations sustaining the structure and composition of the original component. The use of lipophilic dyes for plasma membrane staining will therefore afford additional cytoplasm markers due to endosome formation from the plasma membrane previously stained with the fluorescent compound. There is no way to eliminate this fluorescent signal because of the constitutive presence of endosomes in mammalian cells. Cancer cells have, in addition, an accelerated metabolism and high endocytosis index [63]. Endocytosis is known to be the cellular event that plays a pivotal role in endosome formation and maintenance. The presence of intracellular structures marked with BTD-4APTEG is then explained by endosome formation. The mild fluorescent noise observed over the cells is probably caused by the plasma membrane involving the cells and its fluorescent signal has then contributed to the image formation.

Finally, the developed fluorophore was tested in additional cell lines to show its efficiency. A2780 (human ovarian carcinoma) cells, T47D (human breast tumor) cells and HUVEC (human umbilical vein endothelial) cells were tested and the results are shown in Figure S8 (Supporting Information File 1). Again, the designed fluorophore was capable of selectively stain the plasma membranes with intense green emissions, indicating therefore, the fluorogenic dye efficiency as a new bioprobe for bioimaging experiments.

## Conclusion

In summary, a new water-soluble BTD fluorophore BTD-4APTEG was developed and applied as selective probe for bioimaging and stained plasma membranes selectively in the tested cells lines. The features envisaged for the synthesis of the structure proved to be capable of granting the dye water solubility, good photostability and affinity for the plasma membrane as depicted in the imaging (qualitative and quantitative) experiments. Theoretical calculations were found to be in accordance with the experimental data and helped to understand the ICT stabilizing process of the designed fluorophore. The developed green emitter was efficiently applied as selective plasma membrane probe in bioimaging experiments. Co-staining and PCC experiments confirmed the dye’s affinity for the plasma membrane and indicated its efficiency as a new bioprobe.

## Experimental

NMR spectra were recorded on an NMR instrument using a 5 mm internal diameter probe operating at 400 MHz for ^1^H and at 100 MHz for ^13^C NMR. Chemical shifts were expressed in parts per million (ppm) and referenced by the signals of the residual hydrogen atoms of the deuterated solvent, as indicated in the legends. UV–vis absorption (Varian Cary 5000) spectroscopy and fluorescence emission (Cary Eclipse,Varian CA-USA) were acquired using recent prepared solutions (10 μM for all analyses). All reagents and solvents were purchased from commercial sources.

**Synthesis of BTD-4APTEG.** 2-(2-(2-Methoxyethoxy)ethoxy)ethyl methanesulfonate (48 mg, 0.2 mmol) and BTD-4AP [41] (11.5 mg, 0.05 mmol) were mixed in MeCN (5 mL) in a sealed Schlenk tube and the reaction mixture was stirred at 80 °C for 24 h. After cooling, the solvent was removed and the crude washed several times with ethyl acetate to remove unreacted reagents. The desired product was obtained in 65% yield. IR (cm^−1^): 3090, 2870, 1960, 1630, 1514, 1220, 1106, 930; ^1^H NMR (400 MHz, D_2_O) δ (ppm) 8.15 (d, *J* = 7.2 Hz, 2H), 8.00–7.95 (m, 1H), 7.84–7.65 (m, 2H), 7.13 (d, *J* = 7.2 Hz, 2H), 4.37 (t, *J* = 5.2 Hz, 2H,) 3.88 (t, *J* = 5.2 Hz, 2H,) 3.65–3.44 (m, 8H), 3.21 (s, 3H), 2.74 (s, 3H); ^13^C NMR (100 MHz, D_2_O/CD_3_OD 1:1, v/v) δ (ppm) 156.4, 155.8, 144.1, 130.9, 129.2, 126.4, 123.3, 120.1, 111.9. 71.50, 71.47, 70.2, 69.9, 69.3, 38.9; HRMS (ESI-Q-TOF) calcd. for C_18_H_23_N_4_O_3_S^+^, 375.1485; found, 375.1460.

**Theoretical calculations.** All DFT calculations were performed using the Gaussian 09 suite of programs [64]. Geometry optimizations were carried out with the long-range corrected density functional CAM-B3LYP with 6-31G(d) Pople’s split basis set. Harmonic frequency calculations were performed to verify that a genuine energetic minimum was achieved. Solvent effects on the BTD-4APTEG geometries were assessed using the polarizable continuum model (PCM) in which the solute molecule is enclosed in a cavity embedded in a continuum dielectric medium. The optimized geometries of the ground state (S_0_) in the calculated solvents were then used for the single point TD-DFT calculation using density functionals of different flavors to assess the performance of different density functionals: B3LYP, CAM-B3LYP, LC-ωPBE, M06, M06-2X, and PBE1PBE. It was employed the 6-311+G(2d,p) basis set to simulate the excitation spectra of the BTDs. To comprise the solvent effects, the implicit PCM treatment was also included in the TD-DFT calculations.

**Biological experiments.** The new BTD derivative BTD-4APTEG was diluted in water in the cell medium supplemented with 10% of fetal calf serum. The following cell lines were used: human ovarian cancer cell line A2780, human breast adenocarcinoma cell line MCF-7, breast adenocarcinoma cell line T47D and human umbilical vein endothelial cells, HUVEC. The cells were maintained according to ATCC (American type culture collection) recommendations at 37 °C in an atmosphere containing 5% CO_2_.

**Cell viability.** For cell viability the synthesized compound BTD-4APTEG was tested at two different concentrations, 10 and 100 μM. The cells were incubated with the synthesized BTD for 24 h and analyzed by a standard MTT assay, following the manufacturer’s recommendations (R&D System Inc, MN, USA). Briefly, 3 × 10^3^ cells of each cell line were seeded in a 96-well plate and maintained overnight at 37 °C. The samples were incubated with 150 μL of MTT (3-[4,5-dimethylthiazol-2-yl]-2,5-diphenyltetrazolium bromide) solution (0.5 mg mL^−1^) in cell culture medium for 4 h in the dark at 37 °C (MTT is reduced by metabolically active cells to insoluble purple formazan dye crystals that accumulate inside the cell cytoplasm). Afterwards, the MTT solution is removed and 200 μL of DMSO are added to all samples to solubilize the formazan dye crystals. The plate was read in spectrophotometer and the optimal wavelength for absorbance was 570 nm. The MTT assay was performed in triplicate and also made three independent assays. The cell viability inhibition was determined by evaluation of MTT result obtained for test samples compared with the control samples in the same conditions, following the expression: [survival % = [(tested sample-blank)/(control sample-blank)] × 100].

**Bioimaging experiments.** The bioimaging experiments were performed in a similar manner to a procedure which have already been published elsewhere [65]. Cells were seeded on 13 mm round glass coverslips on the bottom of a 24-well plate and allowed to adhere overnight. Afterwards, the cells were washed three times with serum-free medium aiming at removing non-adherent cells. After reaching the expected confluence, the cells were separated in two main samples, that is, live samples and fixed samples. Live cells were therefore incubated for 30 minutes with a BTD-4APTEG solution (1 μM) at 37 °C, washed three times with PBS 1X (pH 7.4) at room temperature and fixed in formaldehyde 3.7% for 30 minutes. Again, the cells were washed three times in PBS 1X (pH 7.4) at room temperature and the coverslips were mounted over glass slides using ProLong Gold Antifade (Invitrogen, OR, USA) according to the manufacturer’s recommendations. A similar procedure was followed for fixed cells. Fixed cells were washed three times in PBS 1X (pH 7.4) and then fixed in formaldehyde 3.7% for 30 minutes. Afterwards, fixed cells were washed three times in PBS 1X (pH 7.4) at room temperature and incubated for 30 minutes with BTD-4APTEG solution (1 μM) at room temperature, washed three times in PBS 1X (pH 7.4) at room temperature and the coverslips were mounted over glass slides using ProLong Gold Antifade (Invitrogen, OR, USA) according to the manufacturer’s recommendations. These two main samples (of live and fixed cells) were analyzed using confocal microscopy and excited using 405 nm wavelength laser emission and the fluorescence images were acquired at 520–550 nm wavelength range. Triplicated assays could be carried out and the procedure was performed as three repetitions for each experimental condition.

**Plasma membrane co-staining.** The cell membrane staining procedures were performed with CellMask, a specific fluorescent commercial marker indicated to membrane staining. Briefly, live and pre-fixed cells (processed as described above) were incubated with a CellMask solution (prepared according the manufacturer’s instructions) or with BTD-4APTEG during 30 minutes at room temperature. Afterwards, the cells were washed three times in PBS and the samples were mounted over glass slides by using antifade agent Prolong Gold (Invitrogen, OR, USA) according to the manufacture’s recommendations. The samples were analyzed using confocal microscopy. CellMask was excited at 633 nm wavelength and the fluorescent images were acquired at 680–720 nm wavelength range. BTD-4APTEG was excited at 405 nm wavelength and the fluorescence images were acquired at 520–550 nm wavelength range. All assays were performed in triplicate and it was done three repetitions for each cell sample and experimental condition.

## Supporting Information

File 1 Copies of spectra, additional figures, energies and Cartesian coordinates for all calculated structures.
