# Supplementary material for: Plasma membrane imaging with a fluorescent benzothiadiazole derivative
Source: Beilstein J Org Chem. 2019 Nov 6;15:2644–54. doi: 10.3762/bjoc.15.257 (PMC6880836; doi:10.3762/bjoc.15.257)
Supplement: File 1 — Copies of spectra, additional figures, energies and Cartesian coordinates for all calculated structures. [file Beilstein_J_Org_Chem-15-2644-s001.pdf]

BEILSTEIN JOURNAL OF ORGANIC CHEMISTRY

Supporting Information

for

Plasma membrane imaging with a fluorescent benzothiadiazole derivative

Pedro H. P. R. Carvalho, Jose R. Correa, Karen L. R. Paiva, Daniel F. S. Machado, Jackson D. Scholten and Brenno A. D. Neto

Beilstein J. Org. Chem. **2019,** *15,* 2644–2654. doi:10.3762/bjoc.15.257

Copies of spectra, additional figures, energies and Cartesian coordinates for all calculated structures

License and Terms: This is a supporting information file under the terms of the Creative Commons Attribution License (http://creativecommons.org/licenses/by/4.0). Please note that the reuse, redistribution and reproduction in particular requires that the authors and source are credited.
The license is subject to the *Beilstein Journal of Organic Chemistry* terms and conditions: (https://www.beilstein-journals.org/bjoc)

Table of contents

Figure S1 Page S2

Figure S2 Page S2

Figure S3 Page S3

Figure S4 Page S3

Figure S5 Page S4

Figure S6 Page S4

Figure S7 Page S5

Figure S8 Page S5

Cartesian coordinates of the calculated structure Pages S6–S14

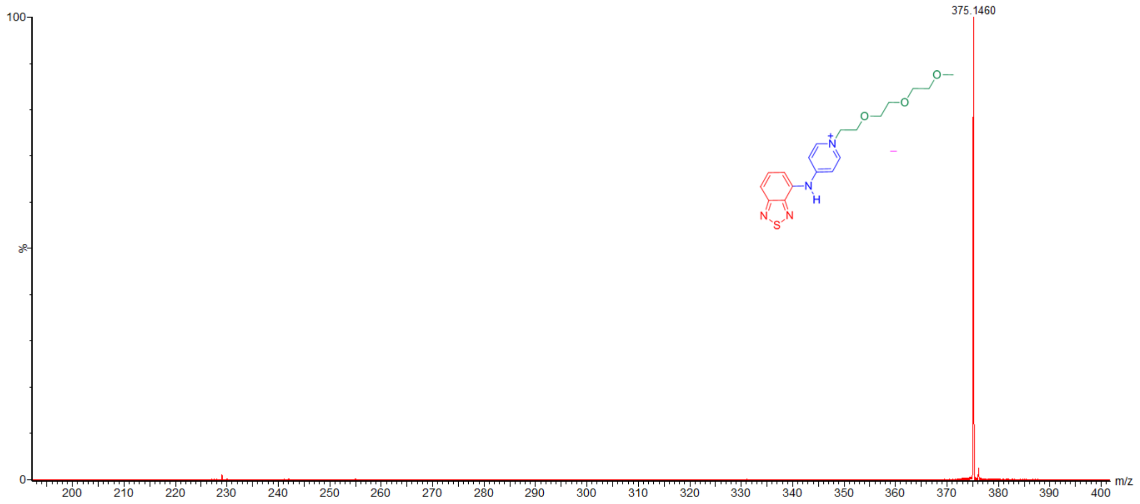

Figure S1: High Resolution ESI(+)-QTOF product ion spectrum of BTD-4APTEG.

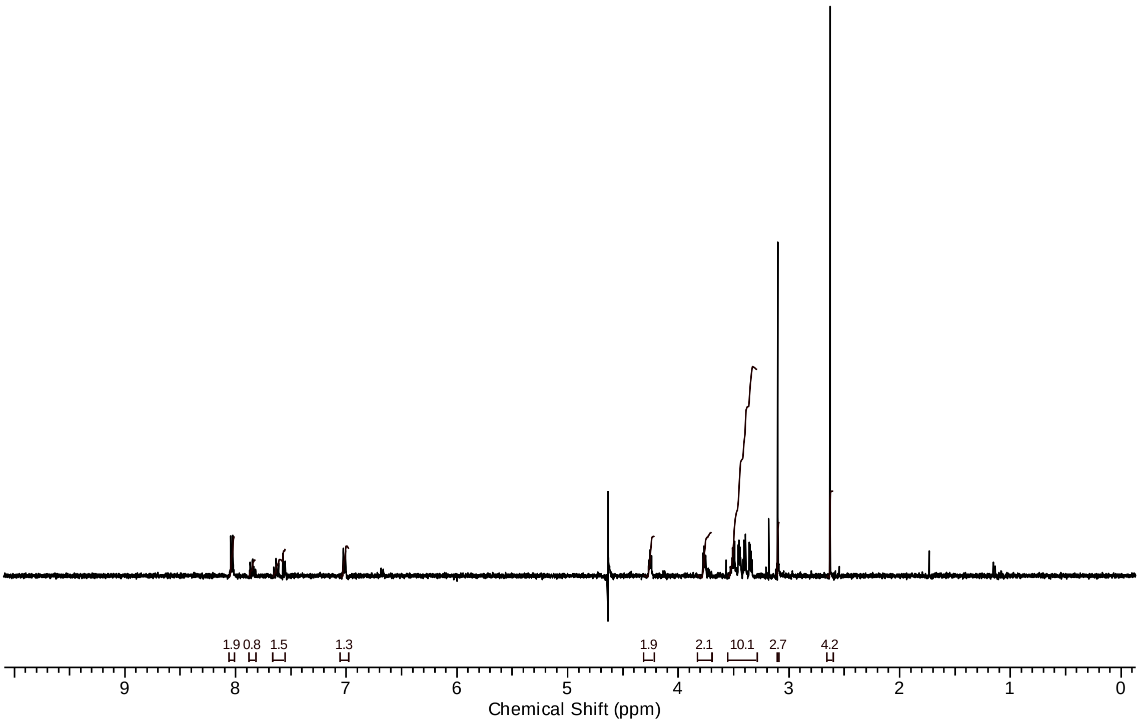

Figure S2: ^{1}H NMR (400 MHz, D_2O) of BTD-4APTEG.

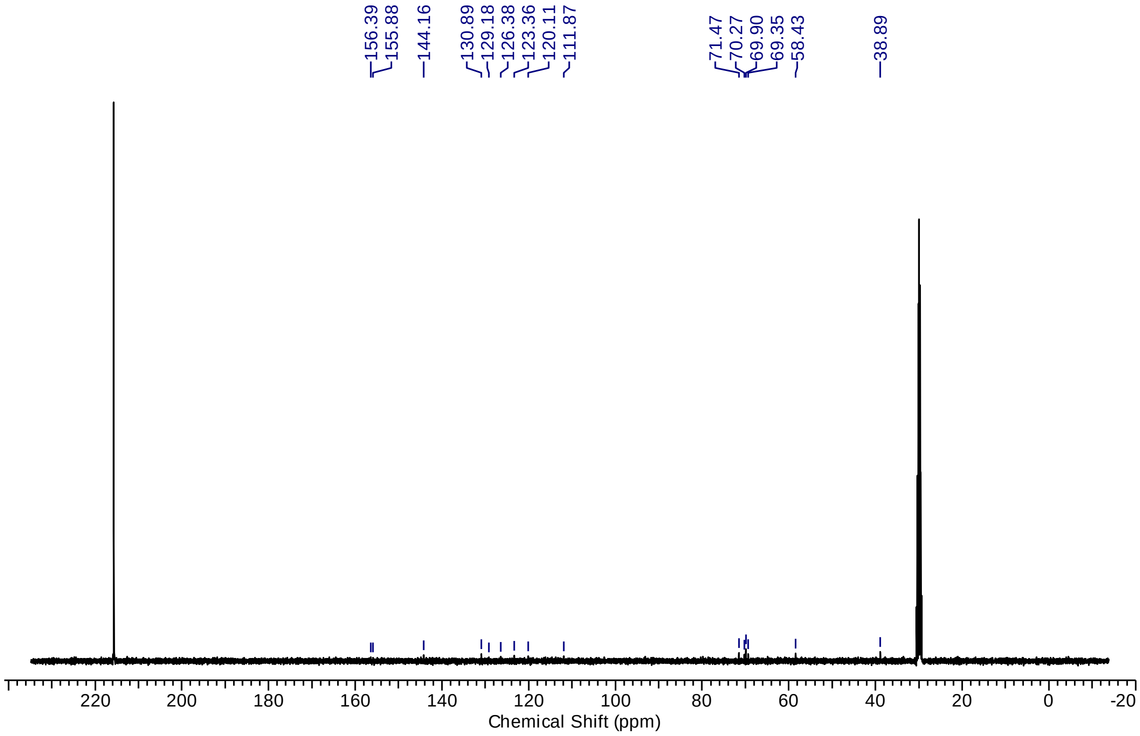

Figure S3: ^{13}C NMR (100 MHz, D_2O/acetone-d_6) of BTD-4APTEG. Deuterated acetone was used to set the scale.

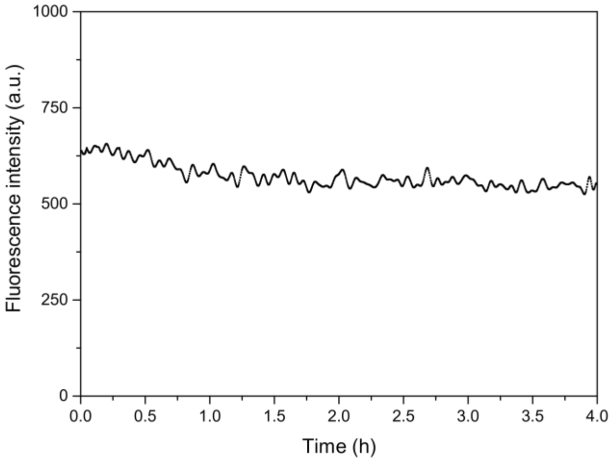

Figure S4: Photostability of BTD-4APTEG acquired at room temperature upon excitation at 254 nm while monitoring the emission at 543 nm. Time-dependence of photoluminescence intensity was carried out with a Xe lamp (400 W).

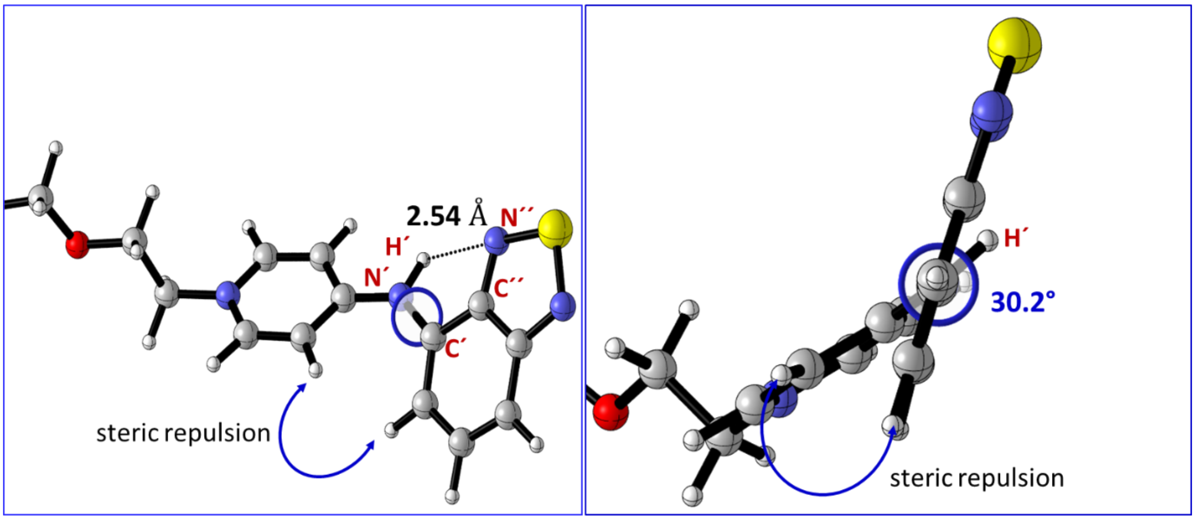

Figure S5: Newman projection of the optimized geometry of BTD-4APTEG in DMSO indicating the distance (black dotted line) between the H’ and the N’’ atoms and the H’-N’-C’-C’’ dihedral angle (blue circle). Results obtained at the CAM-B3LYP/6-311+G(d) level of theory.

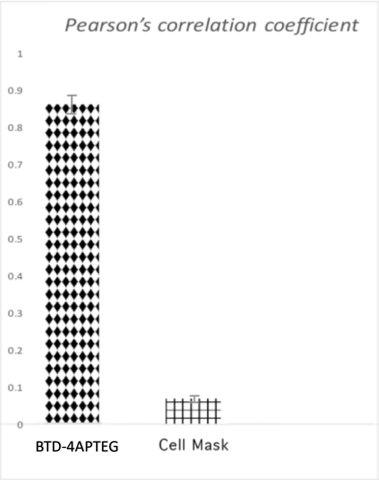

Image Number	Normal	90 degree rotate
1	R = 0.8265	R = 0.0134
2	R = 0.8234	R = 0.0175
3	R = 0.5526	R = 0.0193
4	R = 0.8033	R = 0.0197
5	R = 0.7981	R = 0.0234
6	R = 0.8238	R = 0.0165
7	R = 0.8183	R = 0.0202
8	R = 0.8017	R = 0.0217
9	R = 0.8043	R = 0.0248
10	R = 0.8259	R = 0.0255
	R = 0.79	

Figure S6: PCC from MCF-7 live cells samples (from ten different images) stained with BTD-4APTEG (green emission) and CellMask (red emission). (Left) Pearson’s correlation coefficient of the images with respectively negative controls. (Right) Normal PCC values (average highlighted in yellow) and 90° counterclockwise rotation.

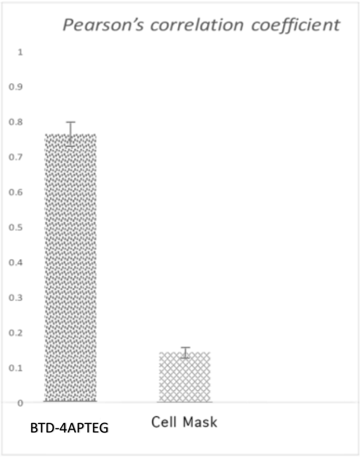

Image Number	Normal	90 degree rotate
1	R = 0.7418	R = 0.2181
2	R = 0.8256	R = 0.1874
3	R = 0.7634	R = 0.2015
4	R = 0.7654	R = 0.2216
5	R = 0.6823	R = 0.1993
6	R = 0.7439	R = 0.2542
7	R = 0.8215	R = 0.2463
8	R = 0.7686	R = 0.2742
9	R = 0.8102	R = 0.2042
10	R = 0.7397	R = 0.2017
	R = 0.77	

Figure S7: PCC from MCF-7 fixed cells samples (from ten different images) stained with BTD-4APTEG (green emission) and CellMask (red emission). (Left) Pearson's correlation coefficient of the images with respectively negative controls. (Right) Normal PCC values (average highlighted in yellow) and 90° counterclockwise rotation.

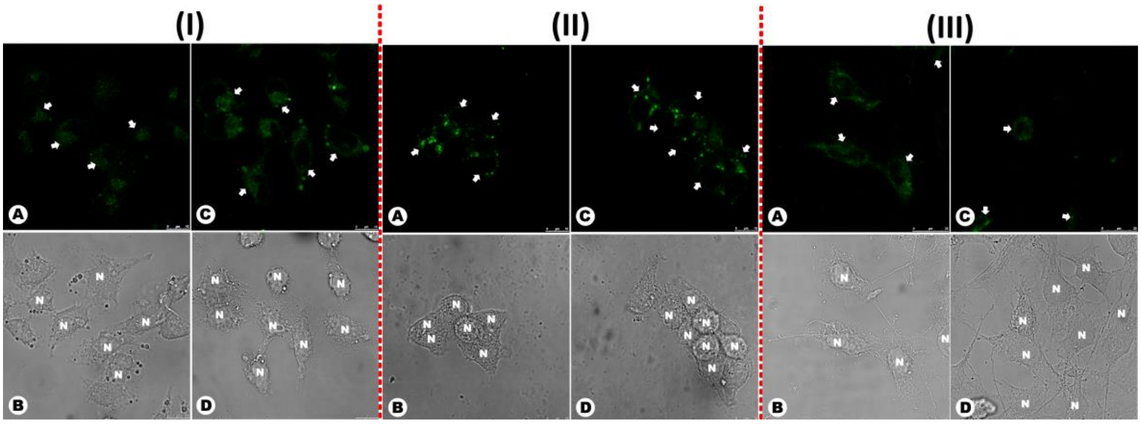

Figure S8: Fluorescent profile of different cell lines incubated with BTD-4APTEG (1 µM) in live (A) and (B) and fixed cells (C) and (D). (I) A2780, (II) T47D and (III) HUVEC cell lines. The dye was found accumulated in the peripheral region of the cellular membrane (white arrows) in both samples. The nuclei in these images are represented by black voids indicated by the letter N. (B) and (D) Show the normal morphological aspects of the samples by phase contrast microscopy. Scale bar of 10 µm.

Cartesian coordinates of the optimized BTD-4APTEG obtained at the CAM-B3LYP/6-31G(d) level of theory.

Energy = -1542.16173568 E_h			
Charge = 1			
Multiplicity = 1			
Solvent = acetonitrile			
C	5.88900600	-0.59196100	0.03641500
C	6.96458200	-0.72111200	-0.90423700
C	7.07064800	0.18109700	-1.99820000
C	6.13827000	1.16501100	-2.09544300
C	5.07626100	1.31596300	-1.14999700
C	4.92119900	0.45043900	-0.10964600
H	7.87651900	0.07794100	-2.71312200
H	6.19501500	1.88046300	-2.90682300
H	4.40631500	2.15774800	-1.26521600
S	7.21520400	-2.43686200	0.73850600
N	7.77767700	-1.73269400	-0.61227000
N	5.91624000	-1.49627200	1.00609400
N	3.92046500	0.52878800	0.87269100
H	4.20528600	0.20841000	1.78879400
C	2.61763300	0.85502400	0.73191000
C	1.81302600	0.96685200	1.88754700
C	1.98708500	1.07120200	-0.50994300
C	0.49802400	1.29566100	1.77665500
H	2.23313000	0.80778100	2.87196600
C	0.66173000	1.38948700	-0.54851500
H	2.51793900	0.97055800	-1.44348000
H	-0.13955200	1.40478600	2.64264400
H	0.14712300	1.55620700	-1.48447800
C	-1.51048000	1.85082400	0.48395900
H	-1.65572200	2.49975700	-0.37743900

H	-1.78387300	2.41304700	1.37475100
N	-0.07954500	1.51601600	0.57261200
C	-2.36500800	0.59956300	0.35826900
H	-2.08626900	0.03131100	-0.53784100
H	-2.22478000	-0.05292500	1.22889600
O	-3.69867300	1.03468400	0.27355800
C	-4.62369500	-0.02692000	0.13120800
H	-4.56050100	-0.70428900	0.99070100
H	-4.40471900	-0.60207000	-0.77586600
C	-6.00578900	0.57983500	0.04311700
H	-6.06158600	1.26565000	-0.81103900
H	-6.22600900	1.15116100	0.95311100
O	-6.92700800	-0.47729600	-0.11149100
C	-8.26520400	-0.03609600	-0.20712300
H	-8.38477100	0.63705700	-1.06480800
H	-8.55192400	0.51263200	0.69828000
C	-9.14386200	-1.25483200	-0.37699800
H	-8.85532200	-1.80416400	-1.28220900
H	-9.02325700	-1.92911300	0.48045000
O	-10.47969500	-0.81342000	-0.47308100
C	-11.39521600	-1.87544000	-0.63694700
H	-12.39109400	-1.44082500	-0.70192200
H	-11.18741000	-2.43768100	-1.55427200
H	-11.35791800	-2.56382600	0.21483900

Energy = -1542.15402740 E_h

Charge = 1

Multiplicity = 1

Solvent = dichloromethane

C	5.88413200	-0.59373300	0.03802000
C	6.95882800	-0.72727800	-0.90250800
C	7.07204300	0.17834900	-1.99292800
C	6.14758300	1.16993300	-2.08729600
C	5.08607300	1.32463100	-1.14183600

C	4.92452600	0.45569600	-0.10554500
H	7.87810800	0.07159500	-2.70703200
H	6.21105800	1.88844900	-2.89548100
H	4.42229400	2.17207100	-1.25274400
S	7.19751100	-2.44892400	0.73464000
N	7.76459000	-1.74537800	-0.61341200
N	5.90458300	-1.50045900	1.00569900
N	3.92303100	0.53823600	0.87746200
H	4.20668600	0.21376800	1.79261900
C	2.62114200	0.86199900	0.73413600
C	1.81272500	0.97455000	1.88776000
C	1.99275100	1.07686400	-0.50981200
C	0.49785000	1.30179700	1.77312000
H	2.23085400	0.81824500	2.87355700
C	0.66696700	1.39218100	-0.55182400
H	2.52719300	0.97616100	-1.44144700
H	-0.14146800	1.41256400	2.63777000
H	0.15354600	1.55603700	-1.48910100
C	-1.50940700	1.85052800	0.47461800
H	-1.65503200	2.49429700	-0.39055100
H	-1.78590500	2.41809900	1.36109800
N	-0.07771400	1.51926000	0.56733800
C	-2.36277200	0.59760600	0.35518200
H	-2.08272800	0.02445500	-0.53785700
H	-2.22092600	-0.05084600	1.22911400
O	-3.69471400	1.03222000	0.26875600
C	-4.62197000	-0.02858700	0.12948400
H	-4.55959900	-0.70393300	0.99055700
H	-4.40468200	-0.60623200	-0.77637700
C	-6.00331500	0.57967900	0.04063300
H	-6.05756200	1.26533100	-0.81392800
H	-6.22248900	1.15254400	0.95008700
O	-6.92374600	-0.47683700	-0.11307800

C	-8.26248300	-0.03660600	-0.20649900
H	-8.38434600	0.63702000	-1.06348600
H	-8.54878400	0.51107400	0.69963400
C	-9.14078900	-1.25556700	-0.37602400
H	-8.85327800	-1.80362600	-1.28249000
H	-9.01719400	-1.93090500	0.48035300
O	-10.47593200	-0.81382300	-0.46841200
C	-11.39251700	-1.87383800	-0.63302200
H	-12.38794800	-1.43777500	-0.69448000
H	-11.18792600	-2.43401400	-1.55254500
H	-11.35455100	-2.56490000	0.21678800

Energy = -1542.16236356 E_h

Charge = 1

Multiplicity = 1

Solvent = DMSO

C	5.88941100	-0.59179200	0.03629100
C	6.96503900	-0.72070700	-0.90438000
C	7.07046000	0.18104000	-1.99878600
C	6.13738700	1.16426200	-2.09641300
C	5.07534900	1.31502400	-1.15097500
C	4.92088500	0.44996400	-0.11012900
H	7.87630200	0.07809100	-2.71377700
H	6.19353000	1.87932000	-2.90818000
H	4.40484800	2.15628000	-1.26669500
S	7.21673600	-2.43568600	0.73910600
N	7.77877400	-1.73167500	-0.61202800
N	5.91725800	-1.49573800	1.00628400
N	3.92024800	0.52808000	0.87217000
H	4.20518700	0.20810600	1.78836700
C	2.61733400	0.85453300	0.73163400
C	1.81309600	0.96635600	1.88746700
C	1.98655700	1.07077400	-0.51002500
C	0.49807900	1.29528700	1.77693100

H	2.23340400	0.80708100	2.87175800
C	0.66123700	1.38931100	-0.54826500
H	2.51706200	0.97008200	-1.44374100
H	-0.13933300	1.40428400	2.64304400
H	0.14649300	1.55623400	-1.48410500
C	-1.51058900	1.85090400	0.48478500
H	-1.65584100	2.50028300	-0.37627800
H	-1.78371200	2.41264800	1.37596000
N	-0.07970600	1.51583600	0.57305000
C	-2.36517900	0.59976100	0.35857900
H	-2.08653100	0.03192100	-0.53778400
H	-2.22506500	-0.05305300	1.22893300
O	-3.69899800	1.03489300	0.27400500
C	-4.62381800	-0.02679300	0.13140400
H	-4.56055000	-0.70432000	0.99077300
H	-4.40468600	-0.60173900	-0.77576300
C	-6.00598100	0.57982200	0.04335800
H	-6.06190800	1.26562900	-0.81078300
H	-6.22629800	1.15104000	0.95338100
O	-6.92725600	-0.47737000	-0.11130500
C	-8.26540600	-0.03609700	-0.20718600
H	-8.38475400	0.63696500	-1.06497300
H	-8.55218300	0.51277500	0.69811600
C	-9.14409100	-1.25482200	-0.37703900
H	-8.85544000	-1.80432300	-1.28210000
H	-9.02377000	-1.92896000	0.48054600
O	-10.47997300	-0.81342900	-0.47350200
C	-11.39541600	-1.87561600	-0.63726600
H	-12.39132100	-1.44111000	-0.70259800
H	-11.18731800	-2.43809200	-1.55436200
H	-11.35821300	-2.56372200	0.21473100

Energy = -1542.16148748 E_h

Charge = 1

Multiplicity = 1

Solvent = methanol

C	5.88884500	-0.59202700	0.03646500
C	6.96440100	-0.72127800	-0.90417900
C	7.07071900	0.18110600	-1.99797500
C	6.13861400	1.16529000	-2.09507300
C	5.07661600	1.31632300	-1.14962400
C	4.92132100	0.45062500	-0.10946100
H	7.87660000	0.07786300	-2.71286900
H	6.19559400	1.88089100	-2.90630600
H	4.40688700	2.15831400	-1.26465200
S	7.21460100	-2.43731900	0.73828200
N	7.77724400	-1.73310000	-0.61235800
N	5.91584000	-1.49647500	1.00602700
N	3.92055100	0.52907200	0.87289200
H	4.20532700	0.20854000	1.78895900
C	2.61775100	0.85522200	0.73201600
C	1.81300000	0.96705800	1.88757600
C	1.98729100	1.07137000	-0.50991400
C	0.49800500	1.29581900	1.77654400
H	2.23302500	0.80807200	2.87204500
C	0.66192300	1.38955500	-0.54861600
H	2.51828100	0.97074000	-1.44338100
H	-0.13963600	1.40499700	2.64248500
H	0.14736800	1.55619100	-1.48462800
C	-1.51043700	1.85079500	0.48363200
H	-1.65567700	2.49955000	-0.37789900
H	-1.78393600	2.41320900	1.37427200
N	-0.07948100	1.51609200	0.57243700
C	-2.36493900	0.59948800	0.35815000
H	-2.08616400	0.03106900	-0.53786000
H	-2.22466600	-0.05287000	1.22888600
O	-3.69854400	1.03460200	0.27338300

C	-4.62364400	-0.02697100	0.13113200
H	-4.56048000	-0.70427500	0.99067600
H	-4.40472900	-0.60220200	-0.77590300
C	-6.00571200	0.57983900	0.04302200
H	-6.06145800	1.26565400	-0.81114400
H	-6.22589400	1.15121100	0.95300200
O	-6.92690900	-0.47726900	-0.11156100
C	-8.26512300	-0.03609800	-0.20710000
H	-8.38477300	0.63708500	-1.06475000
H	-8.55182100	0.51258100	0.69833800
C	-9.14377100	-1.25483900	-0.37697700
H	-8.85527100	-1.80411200	-1.28224200
H	-9.02305600	-1.92917000	0.48042300
O	-10.47958400	-0.81341800	-0.47291800
C	-11.39513600	-1.87537400	-0.63681700
H	-12.39100200	-1.44071400	-0.70165900
H	-11.18744300	-2.43752900	-1.55422800
H	-11.35780400	-2.56386400	0.21489100

Energy = -1542.16317686 E_h

Charge = 1

Multiplicity = 1

Solvent = water

C	5.88994800	-0.59156000	0.03613300
C	6.96563200	-0.72019800	-0.90457000
C	7.07019100	0.18089300	-1.99959600
C	6.13619500	1.16319600	-2.09776200
C	5.07412600	1.31373900	-1.15233600
C	4.92046600	0.44933500	-0.11079700
H	7.87599000	0.07819300	-2.71467900
H	6.19153600	1.87769900	-2.91007100
H	4.40288300	2.15428200	-1.26875100
S	7.21877100	-2.43408700	0.73996600
N	7.78022000	-1.73033800	-0.61167600

N	5.91861700	-1.49498000	1.00657800
N	3.91996500	0.52717400	0.87145900
H	4.20507000	0.20775500	1.78778600
C	2.61694400	0.85390800	0.73125000
C	1.81320300	0.96575600	1.88734800
C	1.98585000	1.07019400	-0.51015200
C	0.49816400	1.29484200	1.77728500
H	2.23378800	0.80624200	2.87147000
C	0.66057500	1.38905900	-0.54794900
H	2.51588400	0.96940600	-1.44410600
H	-0.13902700	1.40369100	2.64356300
H	0.14563900	1.55621700	-1.48362600
C	-1.51073600	1.85101100	0.48586900
H	-1.65601200	2.50096300	-0.37476200
H	-1.78350200	2.41213200	1.37754000
N	-0.07992100	1.51561000	0.57361700
C	-2.36540300	0.60001400	0.35900700
H	-2.08686900	0.03270100	-0.53767500
H	-2.22543600	-0.05320600	1.22901700
O	-3.69942300	1.03515900	0.27460200
C	-4.62398100	-0.02663600	0.13168000
H	-4.56061900	-0.70436200	0.99089500
H	-4.40464100	-0.60132500	-0.77560300
C	-6.00623000	0.57979600	0.04368000
H	-6.06232300	1.26558100	-0.81044900
H	-6.22667700	1.15088500	0.95373400
O	-6.92757900	-0.47747600	-0.11104300
C	-8.26566700	-0.03610400	-0.20727600
H	-8.38471900	0.63681400	-1.06521800
H	-8.55252500	0.51298200	0.69787400
C	-9.14439200	-1.25481600	-0.37707500
H	-8.85558900	-1.80456700	-1.28191700
H	-9.02445900	-1.92874100	0.48071200

O	-10.48033200	-0.81344100	-0.47406600
C	-11.39568200	-1.87584100	-0.63767900
H	-12.39161800	-1.44147100	-0.70350200
H	-11.18719400	-2.43865200	-1.55445700
H	-11.35862000	-2.56355700	0.21461400
